# Development of a Mass Antiviral Screening System Using Viral Hemorrhagic Septicemia Virus as an RNA Surrogate and Activity Confirmation with a Fish Rhabdovirus

**DOI:** 10.3390/v17111522

**Published:** 2025-11-20

**Authors:** Ji Woo Shin, Su Yeon Kim, Min Jeong Kim, Taek-Kyun Lee, Tae-Jin Choi

**Affiliations:** 1 Department of Microbiology, School of Marine and Fisheries Sciences, Pukyong National University, Busan 48513, Republic of Korea; sjw3003@gmail.com (J.W.S.); gsuyeon634@gmail.com (S.Y.K.); 2 Risk Assessment Research Center, Korea Institute of Ocean Science & Technology, Geoje 53201, Republic of Korea; min-jeong214@kiost.ac.kr (M.J.K.); tklee@kiost.ac.kr (T.-K.L.)

**Keywords:** antiviral agent, surrogate virus, VHSV, screening system

## Abstract

With emerging viruses and drug resistance on the rise, the discovery and development of innovative antiviral substances and agents are necessary for the effective treatment and control of viral outbreaks. Surrogate viruses are safer alternatives used in research to mimic dangerous or hard-to-culture viruses. They enable efficient, ethical, and cost-effective screening of antiviral compounds. In this study, we used a recombinant viral hemorrhagic septicemia virus (rVHSV) expressing enhanced green fluorescent protein as a surrogate for RNA viruses for the high-throughput screening of antiviral agents. An optimized mixture of viruses and EPC host cells was distributed in 96-well plates containing chemical compounds or plant extracts for screening. Using this system, 44,642 chemical compounds and 8104 plant and marine organism extracts were tested; 140 candidates were selected from primary screening, and 8 compounds and 5 plant extracts were further selected based on the selectivity index (SI), representing the ratio of the cytotoxic concentration (CC_50_) to the inhibition concentration (IC_50_). Among these, compound **3**, which had the highest SI value of 1046, was further tested, considering in vitro activity against VHSV and another fish rhabdovirus, snakehead rhabdovirus (SHRV).

## 1. Introduction

The emergence of zoonotic diseases, such as Middle East respiratory syndrome (MERS) and coronavirus disease 2019, threatens human health. Most viruses, including marine ones, are host-specific, and so infection is usually limited to a single species. However, viruses acquire the ability to infect other species or evade vaccine-induced immunity via mutation and reassortment [1,2]. Such genetically modified viruses are continuously emerging, and the spread of the diseases for which they are responsible is accelerated by intensive agriculture, international travel, and climate change [3,4]. Vaccines and antiviral drugs can prevent and treat viral diseases, but vaccines may be ineffective or unsuitable due to rapid viral mutations and the variability of host immune responses [5,6,7], necessitating the development of novel vaccines, which would preferably protect against multiple related viruses. Antiviral drugs act by intervening in the replication cycle of a virus, rather than destroying it [8]. Since the first antiviral agent, idoxuridine, was introduced in 1963, more than 100 antiviral drugs have received regulatory approval, including lopinavir/ritonavir for HIV and favipiravir for influenza [9,10]. These have been used to treat viral infections for decades, saving countless lives [7]. However, most of the currently available antiviral drugs exhibit limited activity, being effective only against a single viral species or a few specific genotypes, resulting in a very narrow spectrum of application [10,11]. Therefore, the development of broad-spectrum antiviral drugs that can act against various strains is necessary to respond quickly to newly emerging viruses.

Efforts are underway to identify antiviral agents using, for example, target-specific methods that inhibit function by determining target enzymes or nucleic acids [12,13]. Mass screening is expensive, labor-intensive, and time-consuming. By contrast, high-throughput screening (HTS) enables the rapid screening of a large number of samples in a semi-automated manner [14]. In addition, HTS can be applied to plaque, fluorescence, and cell-based assays [15,16,17].

Viruses that require processing above biosafety level 3, such as SARS-CoV and Ebola virus, pose a high risk to humans, hampering the development of antiviral drugs [18]. Expensive and access-limited biosafety facilities are needed to handle such viruses. Due to these difficulties, recent research on antivirals and viral replication mechanisms has used surrogate viruses that mimic the behavior or structure of target pathogens but have high biological safety [19,20]. For example, bovine viral diarrhea virus and cutthroat trout virus have been employed as surrogates for hepatitis C and E viruses, respectively [21,22].

Viral hemorrhagic septicemia virus (VHSV) is a fish-infecting negative-sense RNA virus in the *Rhabdoviridae* family, which includes members that infect not only fish but also humans and other animals, such as the zoonotic rabies virus and vesicular stomatitis virus (VSV). VHSV causes hemorrhagic septicemia in fish such as *Paralichthys olivaceus*. It was first isolated from freshwater-cultured rainbow trout (*Oncorhyuchus mykiss*) in 1963 [23], and it has since been isolated in Europe, North America, and Japan [24,25,26]. Due to its wide host range and geographic distribution, VHSV is defined as a notable fish pathogen by the World Organization for Animal Health, and it frequently causes mass mortality in aquaculture and significant economic losses [27].

There are huge differences among different RNA viruses in their structure, replication cycles and responses to the host immune system, which make the development of broad-range anti-RNA virus drugs difficult. However, VHSV shares key replication features with many medically important RNA viruses, including polymerase-dependent genome transcription, nucleoprotein-encapsidated RNA, and conserved steps in viral assembly [28]. These similarities allow screening campaigns to identify compounds that interfere with core viral replication mechanisms or host pathways broadly used by RNA viruses. VHSV can be efficiently propagated in simple fish cell lines and engineered to express fluorescent or luciferase reporters, enabling rapid, quantitative, and automation-compatible readouts suitable for large libraries [29]. Compared with human viruses requiring BSL-2/3 containment, VHSV can typically be handled under lower biosafety conditions, reducing operational burden, cost, and time. Moreover, its robust cytopathic effect (CPE) allows straightforward primary phenotypic screening.

In this study, we developed an HTS system to discover broad-spectrum drugs against RNA viruses using a recombinant virus expressing eGFP as an RNA surrogate. We optimized the incubation temperature, FBS concentration, and multiplicity of infection (MOI). For the first step, screening 44,642 compounds and 8104 extracts, the intensity of eGFP fluorescence emitted by the replication of the recombinant VHSV was measured, and a CPE was observed. The antiviral activity of selected substances was further tested at different concentrations. Final selection was informed by the specificity index (SI), representing the ratio of IC_50_ to CC_50_. The antiviral activity of a selected candidate, named compound **3**, with an SI value of 1046, was further confirmed in vitro, showing significant inhibition of snakehead rhabdovirus (SHRV) replication.

## 2. Materials and Methods

### 2.1. Cells and Virus

Epithelioma Papulosum Cyprini (EPC) cells and recombinant viral hemorrhagic septicemia virus (rVHSV) were provided by Professor Ki-Hong Kim of the Department of Aquatic Life Medicine, Pukyong National University. EPC cells were cultured in Leibovitz’s-15 medium (L-15 medium, Thermo Fisher, Waltham, MA, USA) supplemented with 10% fetal bovine serum (FBS, Thermo Fisher), 100 units/mL penicillin, and 100 μg/mL streptomycin (Thermo Fisher) and incubated at 28 °C [30].

rVHSV contained the eGFP gene between the N and G genes of the VHSV genome [29] and exhibited green fluorescence in infected cells during replication. For the amplification of this virus, EPC cell monolayers were inoculated with rVHSV by replacing the L-15 medium with 10% FBS with L-15 medium containing 2% FBS, 100 units/mL penicillin, 100 μg/mL streptomycin, and rVHSV at an MOI of 0.01 [29,31]. The cells were incubated at 15 °C without further medium change until a cytopathic effect (CPE) was observed. The virus was harvested by centrifuging cell lysate at 3000× *g* for 5 min and stored at −80 °C.

### 2.2. Measurement of Virus Titer

For plaque assays, EPC cells (5 × 10^6^/well) were seeded into a six-well plate and incubated at 28 °C for 2 days. Virus samples were serially diluted 10-fold and 200 µL of diluent was overlaid on the EPC cells. After incubation for 2 h at 15 °C, 2 mL of plaquing medium (0.8% agarose in L-15 medium containing 2% FBS, 100 units/mL penicillin, and 100 μg/mL streptomycin) was overlaid. After incubation for 7 days, the cells were fixed with 4% paraformaldehyde solution (GeneAll, Seoul, Republic of Korea) for 1 h and stained with 10% crystal violet for 12 h. Viral titer was calculated by enumerating plaque-forming units (PFUs).

### 2.3. Exploration of eGFP Fluorescence Wavelength

The excitation and emission wavelength ranges of eGFP are wide, with peaks at 488 and 511 nm, respectively [32,33]. Therefore, the optimal fluorescence wavelength was determined using three excitation and emission wavelengths (Table 1). EPC cells (100 µL; 1.0 × 10^5^/well) and 100 µL of wild-type VHSV (wVHSV) or rVHSV (MOI of 1, 0.1, or 0.01) were mixed, and 200 μL was inoculated into a 96-well plate. The fluorescence intensity from the top of the plate was assessed 1, 3, 5, and 7 days after inoculation using a Varioskan LUX multimode microplate reader (Thermo Fisher).

### 2.4. Optimal MOI and Culture Conditions for Screening

To identify the optimal screening conditions, we evaluated continuous incubation at 28 °C (optimum for EPC growth [34]), continuous incubation at 15 °C (optimum for VHSV replication [29]), and incubation at 28 °C for 24 h followed by inoculation at 15 °C. The final concentrations of FBS in L-15 medium were 2% and 10% (optima for VHSV replication and EPC growth, respectively [35]). EPC cells in the medium (1.0 × 10^5^/well) were mixed with rVHSV at MOIs of 1, 0.1, 0.01, and 0.001, distributed to 96-well plates, and incubated as above. Fluorescence intensity was measured using a microplate reader (excitation, 490 nm; emission, 516 nm) 1, 3, 5, 7, and 9 days after inoculation.

### 2.5. Screening

#### 2.5.1. Substances for Screening

To screen antiviral agents, we obtained 44,642 compounds from the Korea Chemical Bank (KCB, the Korea Research Institute of Chemical Technology, Daejeon, Republic of Korea), 810 extracts from the Freshwater Bioresources culture collection (FBCC) at the Nakdonggang National Institute of Biological Resources (NNIBR, Sangju, Republic of Korea), 2279 extracts from the International Biological Material Research Center (IBMRC) at the Korea Research Institute of Bioscience and Biotechnology (KRIBB, Daejeon, Republic of Korea), 609 extracts from the Marine Bio-Resource Information System (MBRIS) at the National Marine Biodiversity Institute of Korea (MABIK, Seocheon, Republic of Korea), 3700 extracts from the National Institute of Biological Resources (NIBR, Incheon, Republic of Korea), 603 extracts from the Bank of Bioresources from Island and Coast (BOBIC) at the Honam National Institute of Biological Resources (HNIBR, Mokpo, Republic of Korea), 19 extracts from Nature Product Central Bank (NPCB) at the Korea Research Institute of Bioscience and Biotechnology (KRIBB, CheongJu, Republic of Korea), and 84 extracts from the University of the Philippines Tacloban College. The KCB provided compounds at 5 mM in 96-well plates. Extracts were provided as 20 mg of powder in vials, dissolved in 1 mL of 10% DMSO, and 5 µL (500 µg) of each diluted extract was distributed to a 96-well plate.

#### 2.5.2. Screening Procedures

**Figure 1:**
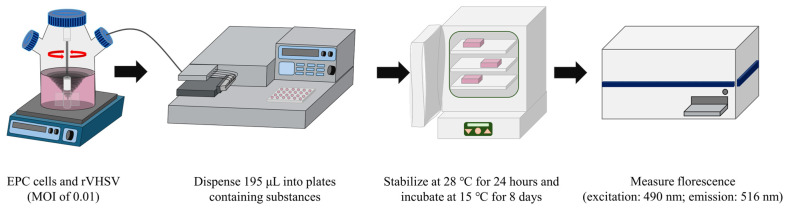
Schematic process for antiviral screening using recombinant VHSV as a surrogate virus. Detailed procedures are described in the text.

A mixture of EPC cells and rVHSV was prepared in neck-suspension culture flasks at an MOI of 0.01 so that the final 200 µL mixture contained 1 × 10^5^ of EPC cells and 1 × 10^3^ PFUs of rVHSV. The flasks were placed on a stirrer (LabTron, Seoul, Republic of Korea) set at 200 RPM, and 195 µL of the mixture was dispensed into each well of 96-well plates containing 5 µL of a compound or extract using a MicroFill microplate dispenser. At this point, the final concentrations of the extracts and the compounds were 125 μM and 500 μg/mL, respectively. After incubation at 28 °C for 24 h, the temperature was shifted to 15 °C. After incubation for 8 days at 15 °C, fluorescence was measured using a Varioskan LUX multimode microplate reader (Thermo Fisher) at excitation and emission wavelengths of 490 and 516 nm, respectively (Figure 1).

### 2.6. Second-Round Screening of Selected Substances

After initial screening of compounds and extracts at 125 μM and 500 μg/mL, respectively, selected candidates were tested at 25, 62.5, and 125 μM (compounds) and 125, 250, and 500 μg/mL (extracts) in a second-round screening. EPC cells (1.0 × 10^5^/well) were incubated for 1 day at 28 °C in 96-well plates. After removing the medium, rVHSV (MOI 0.01) in 195 µL of the same medium was mixed with 5 µL of diluted substance, and the plates were incubated at 15 °C for 7 days. Because a limited number of selected substances were included in the second-round screening, antiviral activity was determined by detecting the CPE using a microscope.

### 2.7. CC_50_ of the Final Candidate

The viability of EPC cells treated with the final candidate was quantified using an EZ-Cytox Kit (DoGenBio, Seoul, Republic of Korea). Briefly, 1.0 × 10^5^ EPC cells/200 µL were incubated at 28 °C for 1 day in a 96-well plate. After removing the medium, 195 µL of L-15 medium containing 10% FBS, 100 units/mL penicillin, and 100 µg/mL streptomycin was mixed with 5 µL of the compound diluted to a final concentration of 100, 200, 400, 600, 800 or 1600 µM; this mixture was added to the wells. The control was 200 µL of L-15 medium with 10% FBS, 100 units/mL penicillin, and 100 µg/mL streptomycin. After incubation at 28 °C for 2 days, the medium was removed and the cells were washed twice with phosphate-buffered saline (pH: 7.2). We added 100 µL of L-15 medium (10% FBS, 100 units/mL penicillin, and 100 µg/mL streptomycin) and 10 µL of water-soluble tetrazolium (WST) solution to the emptied wells and incubated them for 3 h. The absorbance at 450 nm was measured using a Varioskan LUX multimode microplate reader (Thermo Fisher). Cell viability was calculated using the formula recommended by the EZ-Cytox Kit.

$$Viability\;\left(\%\right)=\frac{EXP-blank}{control-blank}\times 100$$

*EXP*: Absorbance of a well with cells, extract, and EZ-Cytox.

*Control*: Absorbance of a well with cells and EX-Cytox, without test solution.

*Blank*: Absorbance of a well with medium and EX-Cytox, without cells.

### 2.8. Determination of the IC_50_ of the Final Candidate Against rVHSV

The IC_50_ values of the final candidate against rVHSV were determined using a plaque assay. EPC cells (200 µL; 1.0 × 10^5^) in L-15 medium (10% FBS, 100 units/mL penicillin, and 100 μg/mL streptomycin) were seeded into wells of a 96-well plate and incubated at 28 °C for 24 h. After removing the medium, rVHSV (MOI 0.01) in 195 µL of L-15 medium supplemented with 2% FBS was mixed with 5 µL of the substance diluted to a final concentration of 16, 8, 4, 2, 1, or 0.5 µM; the mixture was added to the wells. After incubation for 7 days at 15 °C, the supernatant (200 µL) was harvested and subjected to a plaque assay. The results were converted into ratios to the negative control.

### 2.9. Evaluation of Snakehead Virus

To evaluate the broad-spectrum antiviral potential of the selected final candidate (compound **3**) and to validate the applicability of VHSV as an RNA surrogate, inhibition tests were conducted with another fish rhabdovirus, snakehead rhabdovirus (SHRV), of the *Rhabdovirdae* family. SHRV was provided by the laboratory of Professor Ki-Hong Kim of the Department of Aquatic Life Medicine, Pukyong National University [36]. Inhibition against SHRV was tested using plaque assays to determine the viral titer after treatment with compound **3**. EPC cells (200 µL, 1.0 × 10^5^) in L-15 medium (10% FBS, 100 units/mL penicillin, 100 µg/mL streptomycin) were seeded into each well of a 96-well plate and cultured at 28 °C for 24 h. After removing the medium, SHRV (MOI of 0.01) in 195 µL of L-15 medium supplemented with 2% FBS was mixed with 5 µL of substances diluted to final concentrations of 100, 50, 25, 12.5, 4, and 2 µM and added to each well. After incubation for 2–3 days at 28 °C, the supernatant (200 µL) was harvested and subjected to a plaque assay.

To measure the titer of SHRV, plaque assays were performed by seeding EPC cells (5 × 10^6^/well) into 6-well plates and incubating them at 28 °C for 2 days. Virus samples were serially diluted 10-fold and 200 µL of diluent was overlaid on the cells. After incubation for 2 h at 28 °C, 2 mL of plaquing medium (0.8% agarose in L-15 medium containing 2% FBS, 100 units/mL penicillin, and 100 μg/mL streptomycin) was overlaid. After incubation for 2–3 days, the cells were fixed with 4% paraformaldehyde solution (GeneAll) for 1 h and stained with 10% crystal violet for 12 h. Viral titer was calculated by enumerating plaque-forming units (PFUs).

## 3. Results

### 3.1. Optimization of Fluorescence Wavelengths for rVHSV-eGFP

**Figure 2:**
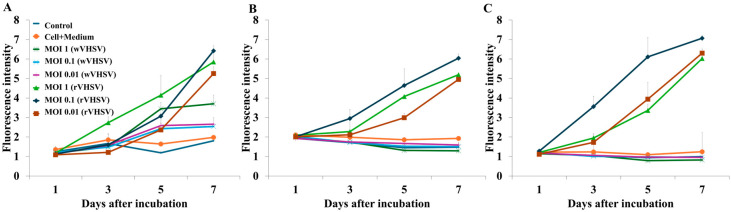
Fluorescence from EPC cells inoculated wVHSV and eGFP-expressing rVHSV at various MOIs. Fluorescence was measured at excitation and emission wavelengths of (**A**) 490 and 516 nm, (**B**) 490 and 516 nm, and (**C**) 480 and 520 nm, respectively. Values are the average of 3 replications, and standard error bars are shown.

In order to determine whether the screening based on eGFP from rVHSV replication was disturbed by autofluorescence from the host cells, autofluorescence from noninfected EPC cells and those inoculated with wVHSV was measured. These cells exhibited high background autofluorescence at excitation and emission wavelengths of 488 nm and 511 nm, respectively, which are known to be the optimum excitation and emission wavelengths of eGFP (Figure 2A). At excitation and emission wavelengths of 490 and 516 nm, respectively, wVHSV and noninfected cells showed low fluorescence intensity compared to cells inoculated with rVHSV. In addition, the fluorescence intensity was slightly higher when the cells were inoculated with rVHSV at an MOI of 0.1 rather than 1 or 0.01 (Figure 2B). Although background fluorescence was absent at excitation and emission wavelengths of 480 and 520 nm, respectively, the fluorescence intensity was lower than that at 490 and 516 nm, respectively (Figure 2B,C). Therefore, excitation and emission wavelengths of 490 and 516 nm, respectively, were used in subsequent experiments.

### 3.2. Optimization of MOI, Temperature, and FBS Concentration for Screening

**Figure 3:**
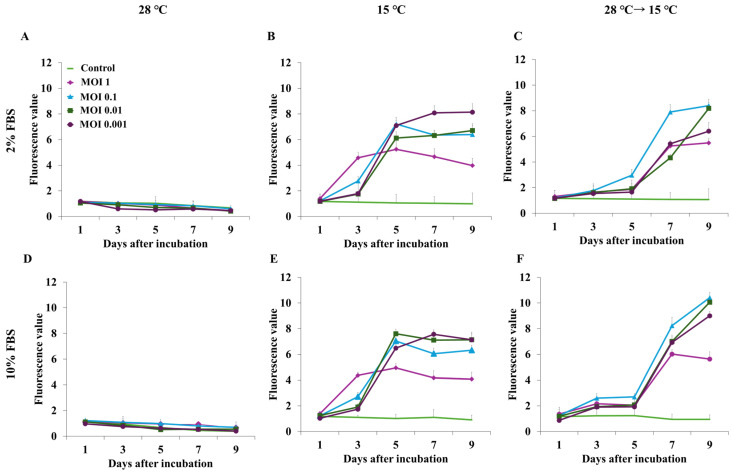
Optimization of incubation temperature, FBS concentration, and MOI for screening of antiviral agents using rVHSV. EPC cells and rVHSV were mixed at the indicated MOIs in L-15 medium containing 2% or 10% FBS and incubated at 28 °C (**A**,**D**) or 15 °C (**B**,**E**) or shifted from 28 °C to 15 °C (**C**,**F**), corresponding to the following conditions. Values are the average of 3 replications, and standard error bars are shown.

EPC cells inoculated with rVHSV at varying MOIs were incubated at 28 °C or 15 °C or shifted from 28 °C to 15 °C one day post-infection using 10% FBS or 2% FBS media, optimal conditions for EPC growth and VHSV replication, respectively. After 9 days of incubation at 28 °C, the fluorescence intensity did not increase regardless of the MOI or FBS concentration, indicating no rVHSV replication (Figure 3A,E). When cells were cultured at 15 °C, the optimal temperature for VHSV replication, the fluorescence increased rapidly after 3–5 days of culture and then plateaued (Figure 3B,F). This pattern was observed at MOIs of 0.1, 0.01, and 0.001 in both 2% and 10% FBS. In contrast, at an MOI of 1, the fluorescence intensity increased after 1 day, peaked on day 3, and then gradually decreased. Finally, when the temperature shifted from 28 °C to 15 °C, fluorescence intensity increased more slowly than that with continuous culture at 15 °C but rapidly increased 4 days after the temperature shift (Figure 3C,F). In this case, fluorescence intensity was higher in the medium containing 10% FBS rather than 2% FBS, except at an MOI of 1, at which it rapidly decreased 6 days after the shift (Figure 3F). Meanwhile, 8 days after the shift, the fluorescence in the cells inoculated at MOIs 0.1 and 0.01 was similar in both 2% and 10% FBS. Therefore, screening was performed at an MOI of 0.01 in 10% FBS and with a temperature shift from 28 °C to 15 °C 1 day after inoculation, followed by 8 days of incubation.

### 3.3. Screening of Antiviral Substances

A total of 44,642 compounds and 8104 extracts were screened for anti-rVHSV activity. Wells with lower fluorescence intensities than those containing EPC cells and rVHSV (negative control) were selected to evaluate cytotoxicity and virus inhibition. The fluorescence intensity of the negative control was approximately 9.0, and wells with lower fluorescence intensities than the negative control were selected and microscopically observed to confirm the CPE. This preliminary screening identified 62 compounds and 78 extracts with lower fluorescence values than the negative control and no CPE.

### 3.4. Second-Round Screening

**Figure 4:**
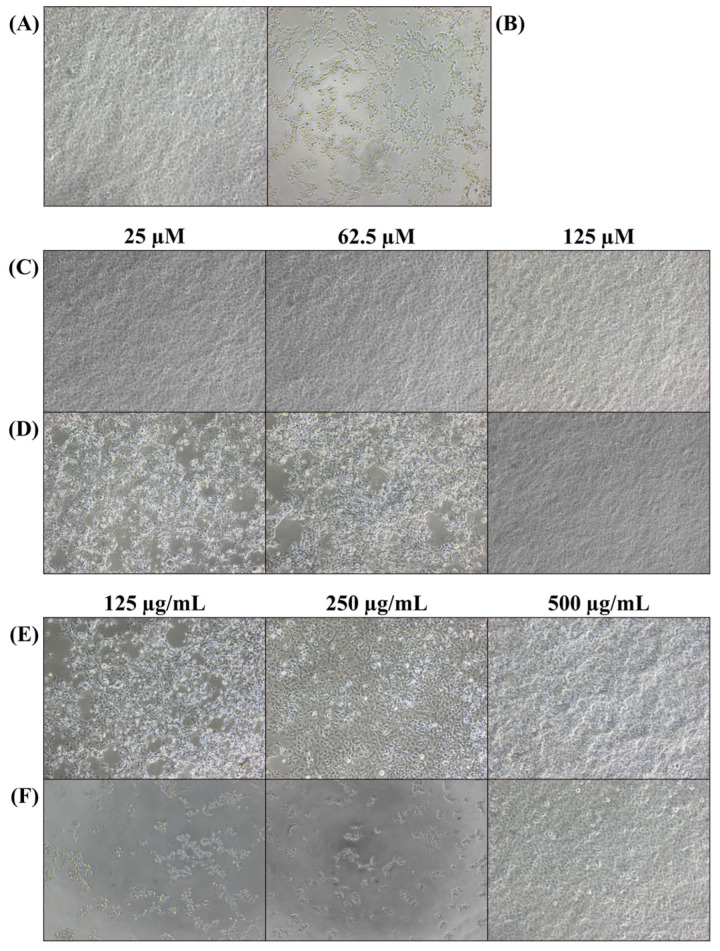
Morphological changes in EPC cells infected with rVHSV and treated with indicated concentrations of the candidate compound and extracts. (**A**) Non-infected positive control. (**B**) Negative control cells were inoculated with rVHSV and without antiviral agents. (**C**) Cells treated with compound **3**. (**D**) Examples of cells treated with 54 excluded chemicals, which showed inhibition at 125 µM. (**E**) Examples of cells treated with 6 selected extracts showing partial inhibition at 250 and 125 μg/mL. (**F**) Examples of cells treated with 72 excluded extracts showing no inhibition at 250 and 125 μg/mL.

Sixty-two compounds and seventy-eight extracts selected in the primary screening were analyzed at different concentrations. Mixtures of EPC cells and rVHSV were treated with the 62 compounds at final concentrations of 25, 62.5, and 125 µM. Among these, eight compounds showed no morphological changes (Figure 4C). Of the other 54 compounds, those that showed no CPE at 125 µM, which was used for primary screening, but clear evidence of CPEs at 62.5 or 25 µM were excluded (Figure 4D). Seventy-eight extracts were tested at final concentrations of 500, 250, and 125 μg/mL. Seventy-three extracts did not show CPE development at 500 µg/mL but showed severe cell lysis at 250 and 125 µg/mL (Figure 4F). Although CPEs were observed in cells treated with the other five extracts at lower concentrations, the cells remained viable (Figure 4E). Based on these results, eight compounds and five extracts were selected as candidates. Among these, one substance, named compound **3**, which showed the best antiviral activity, was selected for further studies. The general name of compound **3** is N-[3-(3-Methyl-1-piperidinyl) propyl]-5-[4-(4-morpholinylsulfonyl) phenyl]-1,3,4-oxadiazole-2-carboxamide.

### 3.5. Cytotoxicity and Antiviral Activity of Compound ***3***

**Figure 5:**
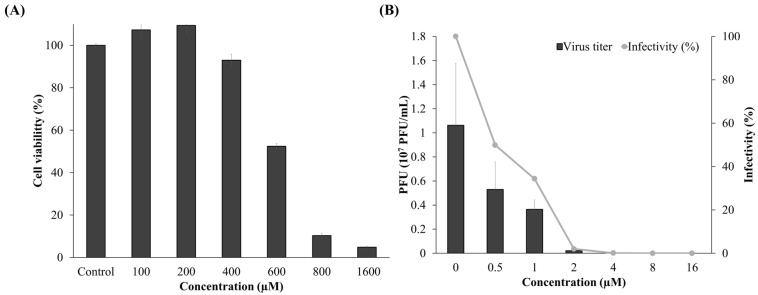
Determination of SI based on cytotoxicity and infectivity testing. The EPC cell line was treated with different concentrations of compound **3**. (**A**) Cell viability at the indicated concentrations was measured using an EZ-Cytox kit for CC_50_ value determination. (**B**) Infectivity was measured by plaque assay for establishing IC_50_ values. Values are the average of 3 replications, and standard error bars are shown.

To determine the SI, the cytotoxicity and antiviral inhibition effects of compound **3** were determined. The CC_50_ values of the final candidate in EPC cells were evaluated using the EZ-Cytox Kit 2 days after treatment. Cell viability decreased in a dose-dependent manner following treatment with compound **3** (Figure 5A). At 600 μM, cell viability decreased to 52%, and at 800 μM, it decreased to nearly 10%, demonstrating significant cytotoxicity at high concentrations. A dose–response analysis using R yielded a CC_50_ value of 585.93 ± 23.15 μM for compound **3**. The IC_50_ of the final candidate for rVHSV was assessed using plaque assays. The number of plaques in cultures treated with compound **3** was divided by that of the negative control and expressed as a percentage. This compound decreased the rVHSV titer in a dose-dependent manner (Figure 5B). At 0.5 and 1 μM, the infectivity was 50% and 34%, respectively. The IC_50_ values of compound **3** were calculated per Sebaugh [37] as 0.56 ± 0.098 μM.

### 3.6. Antiviral Activity Against SHRV

**Figure 6:**
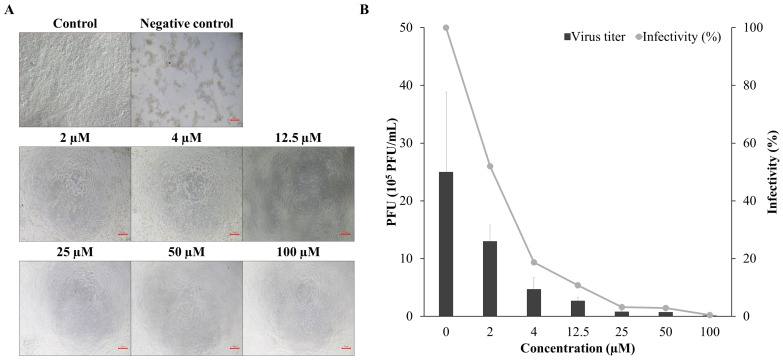
The protective effects of compound **3** against SHRV. EPC cells were challenged with SHRV (MOI of 0.01) with the indicated concentrations of compound **3**. (**A**) Morphological changes 3 days after treatment with compound **3**. (**B**) Viral titer from the supernatant is shown in panel A. Values are the average of 3 replications, and standard error bars are shown.

The antiviral activity of compound **3** was further evaluated against another fish rhabdovirus, SHRV. This candidate was mixed with SHRV prior to inoculation, and mixtures were applied to EPC cells at increasing concentrations. Compound **3** inhibited SHRV-induced cytopathic effects and reduced viral titers in a dose-dependent manner. At concentrations of 12.5 μM or higher, no morphological changes or CPEs were observed, in contrast to outcomes in cells infected with SHRV alone (Figure 6A). Consistent with microscopic observations, compound **3** led to a concentration-dependent reduction in viral titer (Figure 6B). Infectivity was reduced to 52% and 18.7% at 2 μM and 4 μM, respectively. The IC_50_ of compound **3** against SHRV was calculated to be 1.98 ± 0.16 μM. Using a CC_50_ value of 585.93 ± 23.15 μM, the SI value of compound **3** for SHRV was estimated as 294.9.

## 4. Discussion

RNA viruses, including Ebola virus and COVID-19, have caused global pandemics and remain a major threat to human health [38]. Therefore, there is a need to prevent the early stages of outbreaks [39]. Although hundreds of viruses worldwide are known to be pathogenic to humans, currently approved antiviral drugs number only about 100, and even these are effective against only around 10 types of viruses [10]. In other words, most antiviral drugs are designed to treat infections caused by a single virus [8]. To respond rapidly to potential outbreaks of serious infectious viral diseases, the development of broad-spectrum antiviral drugs with high safety and efficacy is essential [40]. These drugs will enable a fast and effective response to public health emergencies, making an important contribution to protecting human life and health.

HTS can rapidly and efficiently screen large libraries (ranging from 100,000 to more than 1 million compounds) using automated equipment [41] for viruses and cells expressing a reporter protein such as an enzyme or fluorescent protein [16,42,43,44]. In particular, the use of viruses expressing fluorescent reporters not only allows easy monitoring of the replication process but also reduces the biosafety level required for research on high-risk pathogens, thereby enabling a more practical approach to antiviral drug development [44,45,46]. Shi et al. [45] developed a recombinant Semliki forest virus expressing eGFP protein (SFV-eGFP), which was used as a surrogate virus for highly pathogenic alphaviruses, which are normally handled in BSL-3 facilities, allowing high-throughput antiviral screening to be safely conducted under BSL-2 conditions. The eGFP-expressing VHSV used in this study can provide direct confirmation of viral inhibition based on the presence or absence of fluorescence.

The maximum excitation and emission wavelengths of eGFP are 488 and 511 nm, respectively [39,40]. In our experiments, cells inoculated with wVHSV exhibited significantly higher autofluorescence compared with uninfected controls (Figure 2A). This suggests a potential risk of nonspecific fluorescence due to cellular autofluorescence [47]. Under the 480/520 nm settings, background fluorescence was minimal; however, the eGFP signal intensity was lower compared with that under the 490/516 nm settings (Figure 2C). In contrast, excitation at 490 nm and emission at 516 nm produced minimal background fluorescence in both wVHSV-infected and uninfected control cells, while yielding the highest eGFP signal in rVHSV-infected cells (Figure 2B). Together, these results demonstrate that excitation at 490 nm and emission at 516 nm represent the optimal conditions for reliable eGFP detection while minimizing cellular autofluorescence.

For general virus amplification, viruses are typically inoculated after cells to form a monolayer [48,49]. In our experiment, it was necessary to dispense the cell-and-virus mixture into 96-well plates by using an automated dispenser for rapid screening of a high number of antiviral substances [49]. The optimum temperature for EPC cell growth is 28 °C, but that for VSV replication is 15 °C [29,30,31]. Optimization of MOI is important in virus replication and inhibitor assays because a high MOI infects all cells simultaneously, resulting in only one replication cycle, rapid CPE development, and lower virus yield [50,51]. Therefore, we optimized the temperature, FBS concentration, and MOI for our rapid screening process by measuring the fluorescence of eGFP, indicating virus replication. As expected, there was no virus replication at 28 °C (Figure 3A,D). When the cells were incubated at 15 °C immediately after inoculation, the fluorescence increased from 3 days after inoculation, reached its maximum around 5 days post-inoculation, and did not increase after that (Figure 3B,E). This indicated that the virus started replication immediately after incubation and lysed the cells even before they formed a complete monolayer, which would not give enough time to evaluate the antiviral activity of the screening materials. Time taken to form a monolayer depends on cell type, initial cell number, and FBS concentration. For EPC cells, it takes 24–48 h to form a monolayer at 28 °C at an initial cell number of 1 × 10^5^ of EPC cells/mL in L-15 medium with 10% FBS [52]. Therefore, after incubation of the EPC cells and rVHSV at 28 °C for 24 h, the temperature was shifted to 15 °C for virus replication. As shown in Figure 3C,F, there was a slow increase in fluorescence, probably due to the temperature being high for the virus but promoting division of cells that were not infected, followed by a faster increase, indicating virus replication. MOI is another significant factor, as a high MOI can result in the rapid death of all of the cells, leaving no more for inoculation. As expected, inoculation of cells at an MOI of 1 resulted in a rapid increase in fluorescence and then a steep decrease, except for a temperature shift from 28 °C to 15 °C in 2% FBS (Figure 3C), due to slow cell growth at this temperature. Furthermore, 8 days after the temperature shift in both 2% and 10% FBS, the fluorescence intensity at MOIs of 0.1 and 0.01, which have a ten-fold difference in virus number, was almost the same, probably because of complete lysis of EPC cells at this stage (Figure 3C,F). A high MOI requires a large initial virus stock but does not guarantee improved yield; thus, a mid-range MOI is recommended to balance production efficiency and viral output [53]. Therefore, the optimum screening conditions were determined as an MOI of 0.01 and a temperature shift from 28 °C to 15 °C after 1 day of incubation in 10% FBS (Figure 3F).

Although previous studies have utilized various surrogate virus models to explore antiviral drugs, systematic screening studies targeting large-scale libraries of tens of thousands or more compounds have rarely been reported [54,55,56,57,58,59]. We screened 44,642 compounds and 8104 extracts for antiviral activity using the HTS system and rVHSV. The 13 final candidate substances (8 compounds and 5 extracts) showed dose-dependent inhibitory effects (Figure 4). Among these candidates, compound **3**, with the highest SI value, was selected as a representative substance to evaluate overall efficacy, and subsequent experiments were conducted.

Compound **3** (C_22_H_31_N_5_O_5_S [MW 478 Da]) inhibited the replication of rVHSV in a dose-dependent manner (Figure 5B). The compound has yet to be fully characterized, and a search of PubChem did not yield reports of its antiviral activity. However, it is known that this compound belongs to the heterocyclic series containing sulfonyl. Notably, sulfonyl- or sulfonamide-based derivatives exhibit potential antimicrobial activity and have been widely investigated for their broad therapeutic effects, including anti-inflammatory and antioxidant properties [60]. In addition, the pharmacological properties of many antiviral drugs (e.g., acyclovir, ribavirin, nelfinavir) involve the presence of heterocyclic rings in their core structures, which provide a structural basis for strong and selective interactions with biological targets [61,62,63].

VHSV, used as an RNA replacement in this study, is an enveloped virus with a negative-strand RNA genome and belongs to the *Rhabdoviridae* family [64]. This family includes the rabies virus, a highly pathogenic zoonotic virus that poses a serious threat to both humans and animals [65]. VHSV has structural similarities to this virus, but it only infects fish and is not transmissible to humans. This disease severely affects a wide range of fish species and causes significant economic losses to the aquaculture industry [66]. Therefore, many studies related to antiviral agents against VHSV have been conducted [67,68,69,70,71]. Kang et al. [72] reported the antiviral activity of fisetin against VHSV, with a CC_50_ value of 500 μM and a half-maximal effective concentration (EC_50_) of 33.3 μM. In addition, Park et al. [68] also observed high inhibition of VHSV proliferation with a mixture of *Celosia cristata* stem and *Raphanus sativus* root extracts at a concentration of 10 µg/mL. In this study, the efficacy of compound **3** was comprehensively evaluated by comparing it with previously reported antiviral agents based on the SI. This compound exhibited potent antiviral activity against VHSV, with an IC_50_ value of 0.56 ± 0.098 μM (Figure 5B). The CC_50_ value was determined to be 585.93 ± 23.15 μM, indicating low cytotoxicity (Figure 5A). Based on these results, the SI was calculated to be approximately 1046. For comparison, Micol et al. [73] reported SI values of 212 and 354 for *Olea europaea* extract and oleuropein against VHSV. In addition, although direct SI values have not been reported for the clinically used antiviral drugs ribavirin and remdesivir, both have been confirmed to have antiviral activity that inhibits viral replication and reduces cell lesions in VHSV-infected cells. Considering these results, the SI value of compound **3** in this study (approximately 1046) is significantly higher than that of existing natural extracts and commercial antiviral agents, and thus, the compound can be evaluated as a promising and safe antiviral candidate against rVHSV.

As the first step for evaluating the possible broad-spectrum antiviral inhibitory effect of compound **3**, it was tested against SHRV, another fish-infecting *Rhabdoviridae*. SHRV was chosen because it shares key structural and biological features with VHSV, including genome organization, replication strategies, and dependence on conserved cellular pathways, which are also relevant to many mammalian negative-strand RNA viruses. Its ability to replicate efficiently at higher, laboratory-compatible temperatures makes it a practical model for evaluating antiviral efficacy [74]. Additionally, SHRV could be propagated in the same EPC cells used for the VHSV experiments, allowing consistent experimental conditions. Testing the candidate compound against SHRV confirmed that its antiviral activity is not restricted to a single virus strain and suggests broader effectiveness across rhabdoviruses, supporting potential application to mammalian RNA viruses [75]. SHRV treated with compound **3** showed CPEs at concentrations below 4 μM, but cell degeneration gradually reduced in a dose-dependent manner (Figure 6A). In addition, it was confirmed that the number of plaques significantly decreased depending on the treatment concentrations of compound **3** (Figure 6B).

In this study, we established a novel HTS platform based on recombinant VHSV-eGFP, enabling semi-automated and reliable detection of viral inhibition through fluorescence readout. VHSV was selected as a surrogate RNA virus due to its safety, its genetic and biological similarity to other negative-strand RNA viruses, and its suitability for high-throughput culture in EPC cells. Although VHSV is widely distributed throughout the Northern Hemisphere, it remains unreported or extremely rare in most regions of the Southern Hemisphere [76]. In this study, we optimized the multiplicity of infection MOI of recombinant VHSV for use in our HTS platform in order to minimize viral input and thereby reduce biosafety concerns. Furthermore, the identification of potent antiviral candidates through this screening effort may facilitate preparedness and support future control measures should VHSV emerge as an economic or ecological threat in previously unaffected regions.

Although the use of a fish rhabdovirus imposes certain limitations on direct extrapolation to mammalian pathogenic RNA viruses due to their extensive diversity, this model remains valuable. Owing to conserved features shared across many RNA viruses—such as the high structural conservation of their RNA-dependent RNA polymerases—the fish rhabdovirus system provides a practical and biosafe platform for early-stage antiviral compound discovery before proceeding to higher-risk mammalian studies. Through screening of 44,642 synthetic compounds and 8104 natural extracts, we identified 13 inhibitors, among which compound **3**—previously not reported to exert antiviral activity—demonstrated concentration-dependent inhibition of VHSV. It also showed cross-reactivity against SHRV and a favorable SI value, supporting its potential as a broad antiviral candidate. Future work will include in vivo testing in fish models, followed by evaluation against mammalian rhabdoviruses and other RNA viruses to identify broad-spectrum antiviral agents.

## Figures and Tables

**Table 1:** Wavelength settings for eGFP measurement using rVHSV-inoculated EPC cells.

No.	Excitation Wavelength (nm)	Emission Wavelength (nm)
1	488	511
2	480	520
3	490	516

## Data Availability

Dataset available on request from the authors.
